# A behavior and physiology-based decision support tool to predict thermal comfort and stress in non-pregnant, mid-gestation, and late-gestation sows

**DOI:** 10.1186/s40104-022-00789-x

**Published:** 2022-12-10

**Authors:** Betty R. McConn, Allan P. Schinckel, Lindsey Robbins, Brianna N. Gaskill, Angela R. Green-Miller, Donald C. Lay, Jay S. Johnson

**Affiliations:** 1 Oak Ridge Institute for Science and Education, Oak Ridge, TN 37830 USA; 2 Department of Animal Sciences, Purdue University, West Lafayette, IN 47907 USA; 3 Department of Agricultural and Biological Engineering, University of Illinois, Urbana, IL 61801 USA; 4 USDA-ARS Livestock Behavior Research Unit, West Lafayette, IN 47907 USA

**Keywords:** Decision support, Gestation, Heat stress, Management, Sows, Thermal index

## Abstract

**Background:**

Although thermal indices have been proposed for swine, none to our knowledge differentiate by reproductive stage or predict thermal comfort using behavioral and physiological data. The study objective was to develop a behavior and physiology-based decision support tool to predict thermal comfort and stress in multiparous (3.28 ± 0.81) non-pregnant (*n* = 11), mid-gestation (*n* = 13), and late-gestation (*n* = 12) sows.

**Results:**

Regression analyses were performed using PROC MIXED in SAS 9.4 to determine the optimal environmental indicator [dry bulb temperature (T_DB_) and dew point] of heat stress (HS) in non-pregnant, mid-gestation, and late-gestation sows with respiration rate (RR) and body temperature (T_B_) successively used as the dependent variable in a cubic function. A linear relationship was observed for skin temperature (T_S_) indicating that T_DB_ rather than the sow HS response impacted T_S_ and so T_S_ was excluded from further analyses. Reproductive stage was significant for all analyses (*P* < 0.05). Heat stress thresholds for each reproductive stage were calculated using the inflections points of RR for mild HS and T_B_ for moderate and severe HS. Mild HS inflection points differed for non-pregnant, mid-gestation, and late gestation sows and occurred at 25.5, 25.1, and 24.0 °C, respectively. Moderate HS inflection points differed for non-pregnant, mid-gestation, and late gestation sows and occurred at 28.1, 27.8, and 25.5 °C, respectively. Severe HS inflection points were similar for non-pregnant and mid-gestation sows (32.9 °C) but differed for late-gestation sows (30.8 °C). These data were integrated with previously collected behavioral thermal preference data to estimate the T_DB_ that non-pregnant, mid-gestation, and late-gestation sows found to be cool (T_DB_ < T_DB_ preference range), comfortable (T_DB_ = T_DB_ preference range), and warm (T_DB_ preference range < T_DB_ < mild HS).

**Conclusions:**

The results of this study provide valuable information about thermal comfort and thermal stress thresholds in sows at three reproductive stages. The development of a behavior and physiology-based decision support tool to predict thermal comfort and stress in non-pregnant, mid-gestation, and late-gestation sows is expected to provide swine producers with a more accurate means of managing sow environments.

## Background

Heat stress (HS) is a threat to swine productivity, health, and welfare [1–3] that will become increasingly common as global temperatures continue to rise and extreme climatic events increase in frequency [4, 5]. Furthermore, the negative effects of global climate change and increasing environmental heat loads on animal agriculture may be exacerbated by advances in genetic selection, nutrition, and management that have increased pig performance leading to more efficient meat production, faster growth rates, and greater lactation outputs by sows to support greater litter sizes [6–8]. While these advancements promote agricultural sustainability and producer profitability, improved performance has resulted in greater overall metabolic heat production in modern swine [9]. This increase in metabolic heat production may be exacerbated by physiological states such as lactation [10, 11] or gestation [12], and reduces the thermal gradient between the pig and the external environment [13]. As a result, HS susceptibility is likely greater, which can negatively impact health and productivity and, subsequently, may reduce the performance, health, and welfare of future generations of pigs through in utero heat stress (IUHS) [3, 14].

Research by our group and others demonstrates that IUHS reduces postnatal productivity, health, and welfare in pigs [3, 14], and has negative implications for swine industry profitability and sustainability. Specifically, IUHS reduces postnatal growth performance [15, 16], increases the prevalence of behaviors indicative of stress (e.g., aggression, lying) during postnatal life [17, 18], exacerbates physiological indicators of stress (i.e., cortisol, ACTH) following common production stressors [18–21], reduces the ability of pigs to maintain euthermia under HS conditions [22, 23], impairs reproductive function [24], and compromises the immune system of pigs during postnatal life [19, 25]. While some studies have unsuccessfully attempted to mitigate the negative effects of IUHS through nutritional strategies [16], pregnant sow management and HS mitigation during gestation likely represents the best method to decrease IUHS incidence and improve the postnatal performance, health, and welfare of pigs gestated during hot times of the year.

The first step in mitigating gestating sow HS and the effects of IUHS in offspring is understanding what environmental conditions cause HS in gestating sows. Currently, there are recommended thermal conditions for swine at different production stages [26]. However, these guidelines are based upon 25- to 41-year-old data estimating the thermal conditions of pigs using mathematical modeling [13] based on previous research [27, 28] rather than animal experimentation. Furthermore, no reproductive stage differentiation exists (i.e., non-pregnant/early gestation vs. mid-gestation vs. late-gestation) [26], which is important because gestation stage impacts sow HS sensitivity [29–31]. In addition, while several researchers have utilized the thermal humidity index (THI) developed by NOAA [32] to predict HS in pigs [33–35], this index was not originally designed for use in pigs, nor does it differentiate HS thresholds based on production stage or physiological state. Thus, THI may not be an accurate and precise predictor of HS in swine. Furthermore, although several swine specific thermal indices or prediction models have been proposed in recent years, these indices rely on the use of theoretical data or predictions [36–38] with validations on a small number (*n* = 8) of animals [39], or limited data collection in a relatively small number of only non-pregnant sows [40], and none to our knowledge have incorporated both behavioral and physiological metrics of thermal stress and thermal comfort in pigs differentiated by reproductive stage. Therefore, the study objective was to develop a swine-specific decision support tool to predict thermal comfort and stress based on the thermoregulatory and behavioral responses of sows with current genetics at three reproductive stages (e.g., non-pregnant, mid-gestation, late-gestation).

## Methods

### Establishing mild, moderate, and severe HS thresholds

#### Thermoregulatory data collection

All live animal data collection procedures were approved by the Purdue University Animal Care and Use Committee (protocol # 1811001823). Animal care and use standards were based upon the *Guide for the Care and Use of Agricultural Animals in Research and Teaching* [41]. All data collection procedures and resulting thermoregulatory, production, and physiological data have been previously presented by McConn et al. [31]. For the purposes of this paper, only the environmental [e.g., dry bulb temperature (T_DB_), dew point (DP)] and thermoregulatory [e.g., skin temperature (T_S_), respiration rate (RR), and body temperature (T_B_)] measures from McConn et al. [31] were considered for the analyses. Briefly, 36 maternal line sows (Yorkshire × Landrace) bred to Duroc sires were tested in 4 repetitions that began and ended at the same approximate time each day. Treatment groups included sows from three reproductive stages: 11 non-pregnant sows (*n* = 2–3/repetition; parity 3.27 ± 0.86; 244.2 kg bodyweight), 13 mid-gestation sows (*n* = 3–4/repetition; 56.38 ± 11.22 d pregnant; parity 3.25 ± 0.83; 218.9 kg bodyweight), and 12 late-gestation sows (*n* = 3/repetition; 97.00 ± 4.95 d pregnant; parity 3.33 ± 0.75; 251.2 kg bodyweight). Early gestation sows were not included as a treatment group because it was expected that their response would be similar to non-pregnant sows due to limited fetal growth in the first trimester, which is the driver of HS sensitivity differences in gestating sows [31]. Sows were moved into individual pens (1.22 m × 2.01 m) in a thermoneutral [TN; 21.1 ± 2.0 °C and 29.4% ± 1.6% relative humidity (RH)] room for 5.0 ± 0.7 d prior to the experiment [31]. At the start of the experiment, sows were moved (approximately 3 m walking distance) [42] into individual pens (1.22 m × 2.01 m) within an environmentally controlled room where they were maintained at the lower end of the currently established TN zone for sows > 100 kg [4] (15.1 ± 1.9 °C and 50.7% ± 5.6% RH) for 270 min prior to the experiment and allowed to acclimate to their new environment. At the time of the experiment, the T_DB_ was increased gradually from 19.84 ± 2.15 °C to 35.54 ± 0.43 °C, over a 400-min period and RH ranged from 32.83% to 50.13% and averaged 40.49% ± 18.57%. The environmental room contained 2 data loggers (Hobo; data logger temperature/RH; accuracy ± 0.20 °C; Onset; Bourne, MA, USA) to record T_DB_, RH, and DP in 5-min intervals as previously described [31]. Room air speed (m/s) was measured with an anemometer (Testo Model 425; Sparta, NJ, USA) at the pig level (approximately 0.50 m above the slatted floor) every 20 min during the entire experiment and measured 0.11 ± 0.10 m/s throughout the trial. Vaginal temperature (referred to as T_B_ in the present paper), T_S_, and RR were measured in 20-min intervals for all sows and measurement methods were previously described by McConn et al. [31]. Briefly, T_B_ was collected using a calibrated thermochron temperature recorder (iButton, calibrated accuracy ± 0.11 °C; resolution = 0.125 °C; Dallas Semi-conductor, Maxim, Irving, TX, USA), T_S_ was measured using an infrared camera (FLIR Model T440, accuracy ± 2%; emissivity = 0.98; resolution = 0.04 °C; FLIR Systems Inc.; Wilsonville, OR, USA), and RR was determined by counting flank movements through visual observation. The DP and T_DB_ that occurred at the exact time T_S_, T_B_, and RR were measured were used to establish the HS thresholds in the analyses. All sows, regardless of reproductive stage, were limit fed to maintenance (2.27 kg/d) resulting in no feed intake differences between sow groups per common commercial swine production practices [43] as previously described [31].

#### Overall regression analyses

**Fig. 1:**
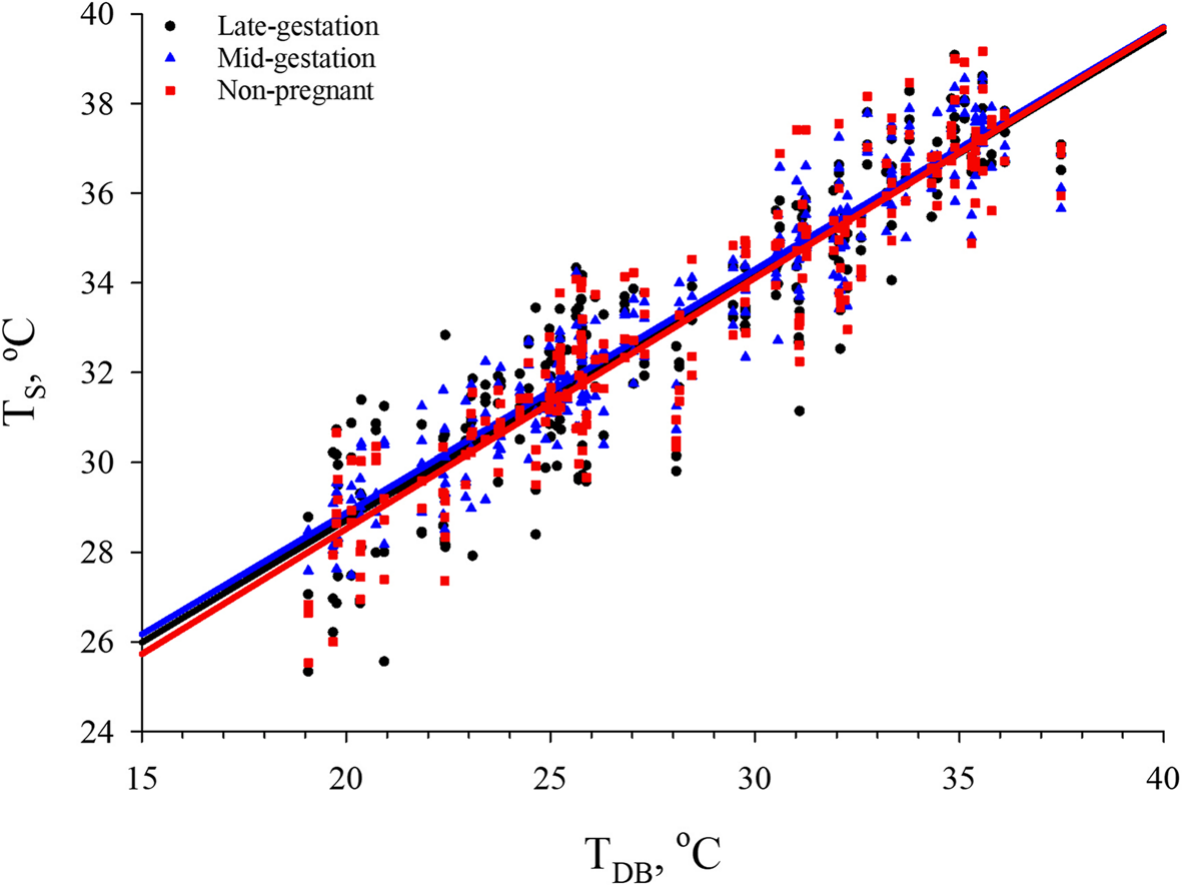
Linear regression of the effects of reproductive stage (late-gestation, mid-gestation, and non-pregnant) on skin temperature (T_S_) in multiparous (3.28 ± 0.81) sows exposed to incrementally increasing dry bulb temperature (T_DB_)

Regression analyses were performed to determine the optimal environmental indicator of HS in non-pregnant, mid-gestation, and late-gestation sows using the linear model procedure with all animal-based indicators successively used as dependent variables. The base model for modeling the animal-based indicators included the effects of T_S_, T_B_, and RR. Skin temperature had a linear relationship with increasing T_DB_, regardless of reproductive stage (Fig. 1) [31]. Therefore, T_S_ was not included in further analyses because this linear relationship was influenced by increasing environmental heat load rather than the sows’ biological HS response or reproductive stage. Regression analyses were then performed, and several combinations of environmental measures (T_DB_ and DP) were added to the model when significant (*P* < 0.05; Table 1). The goodness of fit of the regression equations was evaluated by the Akaike’s Information Criteria (AIC) and residual variance based upon previous research [44–47].

**Table 1:** Evaluation of regression model fit after inflection point determination

Parameter	AIC	Residual variance	*P*-value
Inflection point of T_DB_			
Mild HS			
Late-gestation	1914.00	109.66	0.01
Mid-gestation	1899.00	46.49	0.02
Non-pregnant	1589.50	47.83	< 0.01
Moderate HS			
Late-gestation	–28.90	0.03	0.04
Mid-gestation	–46.20	0.03	0.02
Non-pregnant	76.00	0.06	0.01
Severe HS			
Late-gestation	–28.90	0.03	0.03
Mid-gestation	–46.20	0.03	< 0.01
Non-pregnant	76.00	0.06	0.01
T_DB_ minus T_DB_ breakpoint			
Mild HS			
Late-gestation	1888.80	88.17	0.04
Mid-gestation	1851.40	45.57	0.04
Non-pregnant	1542.20	42.90	0.02
Moderate HS			
Late-gestation	–52.30	0.02	< 0.01
Mid-gestation	–57.80	0.03	0.02
Non-pregnant	42.40	0.05	0.03
Severe HS			
Late-gestation	–41.50	0.01	0.01
Mid-gestation	–69.70	0.02	0.04
Non-pregnant	38.30	0.04	0.04
Inflection point of DP			
Mild HS			
Late-gestation	1918.10	106.99	< 0.01
Mid-gestation	1889.00	44.07	< 0.01
Non-pregnant	ND	ND	0.67
Moderate HS			
Late-gestation	–33.20	0.03	0.01
Mid-gestation	–53.30	0.03	0.02
Non-pregnant	ND	ND	0.21
Severe HS			
Late-gestation	–41.50	0.01	< 0.01
Mid-gestation	–69.70	0.02	< 0.01
Non-pregnant	ND	ND	0.22
DP minus DP breakpoint			
Mild HS			
Late-gestation	1855.60	84.38	0.03
Mid-gestation	1823.90	21.67	0.02
Non-pregnant	ND	ND	0.54
Moderate HS			
Late-gestation	–59.00	0.02	< 0.01
Mid-gestation	–65.60	0.03	< 0.01
Non-pregnant	ND	ND	0.87
Severe HS			
Late-gestation	–66.90	0.01	0.01
Mid-gestation	–70.40	0.01	0.02
Non-pregnant	ND	ND	0.43

*T*_*DB*_ Dry bulb temperature, *HS *Heat stress, *DP* Dew point, *ND* Not determined

#### Separating reproductive stage

The first analysis was conducted using the data set that included the reproductive stages together to evaluate means, variances, and the relationships of RR and T_B_ in sows at different reproductive stages to increasing T_DB_ using the MIXED procedure of SAS (SAS 9.4, Cary, NC, USA) with a heterogeneous AR(1) covariance structure which was selected based on the AIC value (compares the fit of the covariance structures) [48]. Reproductive stage was significant for linear, quadratic, and cubic analyses (*P* < 0.05; Table 2); thus, reproductive stage was separated for future analyses.

**Table 2:** Evaluating the significance of the interaction of reproductive stage and the linear, quadratic, and cubic regression of T_DB_

Parameter	*P*-value
Reproductive stage ×T_DB_	0.04
Reproductive stage × T_DB_ × T_DB_	0.03
Reproductive stage × T_DB_ × T_DB_ × T_DB_	< 0.01

*T*_*DB*_ Dry bulb temperature

#### Random effect of sow

In the second analysis, before separating sow by reproductive stage, the random effect of sow was tested to determine whether it would improve the model, based on the AIC and residual variance. A random effect of sow allows for increased flexibility in fitting the sow variance in the RR and T_B_ curves. Since the addition of sow as a random effect improved the AIC and residual variance (Table 3), the random effect of sow was used for all future analyses.

**Table 3:** Evaluation of regression model fit

Parameter	AIC	Residual variance
Testing sow as random		
Without sow as random		
RR	5855.60	140.51
T_B_	815.20	0.19
With sow as random		
RR	5451.50	67.91
T_B_	5.50	0.04
Testing random effects before inflection point		
Random as B_0_		
RR		
Late-gestation	1962.50	137.98
Mid-gestation	1968.80	66.30
Non-pregnant	1692.50	80.62
T_B_		
Late-gestation	11.00	0.05
Mid-gestation	39.00	0.05
Non-pregnant	110.10	0.08
Random as B_0_ and B_1_		
RR		
Late-gestation	1914.00	109.66
Mid-gestation	1899.00	46.49
Non-pregnant	1589.50	47.83
T_B_		
Late-gestation	–28.90	0.03
Mid-gestation	–46.20	0.03
Non-pregnant	76.00	0.06

*RR* Respiration rate, *T*_*B*_ Body temperature

#### Random effect of TDB

The third analysis was performed to determine how the random effect of T_DB_ would be included to account for the correlation with the individual sow (subject = sow). The random effect was first included as the intercept (B_0_) and then included as B_0_ and linear regression coefficient (B_1_) based on the AIC and residual variance. Since the AIC and residual variance was improved when the random effect was included as B_0_ and B_1_ (Table 3), this was used for the remainder of the analyses. Note, in most cases AIC values are positive; however, in some cases the AIC value can be impacted by an additive constant resulting in a negative AIC value [49].

#### Determination of inflection points

The fourth analysis began by separating the reproductive stage to estimate the environmental measure (T_DB_) as a linear, quadratic, or cubic function for RR and T_B_ based on the AIC and residual variance. These functions were used to describe the sow’s response of RR and T_B_ to changes in the T_DB_. Next, we estimated the inflection points for both variables based on the determined functions and including breakpoint analyses [50]. Specifically, PROC MIXED allowed for inclusion of the random (sow) component in the model with a heterogeneous AR(1) covariance structure to determine the inflection points. Based on the AIC and residual variance (Table 1), T_DB_ minus T_DB_ inflection point was used in the final model. Physiological differences between treatment groups were analyzed using generalized linear mixed models via PROC MIXED with the main effect of the variable (RR or T_B_). A cubic equation was used to describe the RR and T_B_ response due to increasing T_DB_. The inflection point was solved by finding value at which the RR or T_B_ started to increase via breakpoint analyses. The RR inflection point was used to determine mild HS because greater RR is the first active attempt by the sow to dissipate excess heat gain due to the environmental heat load [51, 52]. To determine moderate HS, the T_B_ inflection point was used as an indicator of the T_DB_ at which heat loss mechanisms (i.e., RR and T_S_) could no longer allow the sow to maintain euthermia under a given environmental heat load and the T_B_ set-point was increased above normal as described by Curtis [13]. Finally, an abrupt uncontrollable change in T_B_ (> 0.20 °C) [53] after the inflection point indicated that heat gain overwhelmed heat loss mechanisms and T_B_ began to rise uncontrollably. This was considered the point at which the upper critical temperature limit had been reached and severe HS began to occur as previously described by Curtis [13]. Based on the AIC and residual variance (Table 3), T_DB_ minus T_DB_ inflection point was used for the final analysis. Once the inflection points were detected, the points less than or equal to the inflection point were reanalyzed to confirm there was no linear increase of the variable relative to T_DB_. After reanalysis, it was determined that the points less than or equal to the inflection point had a linear slope equal to zero.

#### Addition of dew point

After mild, moderate, and severe HS thresholds were estimated for T_DB_, DP was added to the model when significant to better fit the model. Dew point represents a true indication of the amount of moisture in the air [54], which was evaluated to determine best fit of the model. The DP inflection points for mild, moderate, and severe HS were determined and were calculated similarly to the T_DB_ inflection points. Based on the AIC and residual variance (Table 1), DP minus DP inflection point was used in the final model. When DP was not significant, DP was removed from the model completely (Table 1). Finally, the cross product of (T_DB_ – T_DB_ inflection point) × (DP – DP inflection point) was also added to the model, based on the improved AIC and residual variance.

### Establishing cool, comfortable, and warm TDB thresholds

The behavioral thermal preference data used to establish cool, comfortable, and warm T_DB_ thresholds in this study were previously collected and published by our group [55]. All live animal data collection procedures were approved by the Purdue University Animal Care and Use Committee (protocol #1712001652). Animal care and use standards were based upon the *Guide for the Care and Use of Agricultural Animals in Research and Teaching* [41]. Briefly, non-pregnant (*n* = 7), mid-gestation (*n* = 5; 58.5 ± 5.7 d pregnant), and late-gestation (*n* = 6; 104.7 ± 2.8 d pregnant) multiparous maternal line (Yorkshire × Landrace; parity 3.4 ± 1.2) sows were selected for testing [55]. Sow thermal preferences were tested using two custom designed thermal gradient apparatuses (12.2 m × 1.52 m × 1.86 m; L × W × H). The thermal gradient apparatuses provided a thermal gradient ranging from 10.35 ± 0.42 °C to 30.49 ± 0.45 °C that was monitored using data loggers (HOBO Data Logger; U12-012, Onset Computer Corporation, MA, USA; temperature range of –20 °C to 70 °C with accuracy of ± 0.35 °C and RH range 5% to 95% with accuracy of ± 2.5% to max 3.5%) placed 0.94 m above the floor and 0.61 m apart [55]. All sows were allowed a 24-h acclimation period within the thermal gradient apparatuses before being tested for an additional 24 h [55]. All sows were allowed to consume their entire daily diet ration (1.82 kg/d) immediately prior to entering the apparatuses per common commercial swine production practices [43] and no reproductive stage-related feed intake differences were observed as previously described [55]. Water was provided ad libitum within the thermal gradient apparatuses. While being housed within the thermal gradient apparatuses, sows were continuously videorecorded and sow location within the apparatuses was compared against T_DB_ measured by the closest data logger [55]. These data were used to generate cubic curves to determine peak T_DB_ preference and the thermal preference range for each reproductive stage [55]. In the present study, thermal preference data generated by Robbins et al. [55] were used to estimate the T_DB_ that non-pregnant, mid-gestation, and late-gestation sows found to be cool (T_DB_ < T_DB_ preference range), comfortable (T_DB_ = T_DB_ preference range), and warm (T_DB_ preference range < T_DB_ < mild HS).

## Results and discussion

Heat stress has well-documented negative effects on the health, productivity, and welfare of sows [31, 56, 57] and their future offspring [3, 14, 58]. As such, a variety of cooling methods have been developed and used in swine facilities to alleviate HS (i.e., floor cooling pads, evaporative cooling pads, chilled drinking water) [10, 59–61]. However, despite the availability and continued development of cooling and management strategies to mitigate HS, recommended or perceived temperature thresholds for implementation may not accurately reflect the thermal requirements of swine. For example, the most recent thermal recommendations for swine by the Federation of Animal Science Societies [41] are based upon 25- to 41-year-old data and likely do not accurately reflect the thermal requirements of swine with current genetics that have been selected for greater litter sizes, lean gain, and have overall greater metabolic heat production [9]. Additionally, these recommendations [26] do not differentiate by sow reproductive stage, which is important because HS sensitivity becomes greater as gestation advances [31, 62, 63]. Although some efforts have been made to develop thermal indices and thresholds for pigs, these efforts have largely focused on the use of theoretical predictions [36–39, 64], have had limited data collection in a relatively small number of non-pregnant sows [40], or have attempted to apply indices originally developed for cattle to pigs [65], and none to our knowledge have differentiated by reproductive stage or used behavioral metrics of thermal preference to identify comfortable temperature ranges for pigs. Therefore, our overall goal was to develop a swine specific decision support tool using both behavioral and thermoregulatory metrics derived from animal experimentation that would provide thermal recommendations for sows at three reproductive stages.

As environmental temperatures begin to rise, cutaneous blood flow increases in an attempt by the body to dissipate excess metabolic heat from the core to the skin where it can be lost to the environment by conductive, convective, evaporative, and radiant heat loss mechanisms [2, 66]. As a result, T_S_ increases as heat gain from the environment becomes greater, and T_S_ has frequently been used as a non-invasive indicator of HS in pigs via the use of thermal imaging cameras [31, 67–69], infrared thermometers [23, 70], and thermocouple probes [71]. Therefore, the relationship between T_S_ and T_DB_ was assessed in the present study to determine whether it could be used as an accurate predictor of thermal stress. However, it was determined that T_S_ had a linear relationship with T_DB_ (Fig. 1) indicating that the increasing environmental heat load (as well as other factors such as air speed) was likely responsible for elevated T_S_ as opposed to the sows’ biological response to HS and total physiological heat load. This observation has implications towards the use of thermal imaging to assess physiological HS in swine. The linear relationship between T_S_ and T_DB_ suggests that thermal imaging and other technologies that assess heat load based upon T_S_ may be useful for determining how T_S_ is directly affected by the environment and is consistent with earlier observations in other species [13]. However, these technologies may not be an accurate or precise method of determining total physiological heat load of an individual pig to assess physiological HS. Therefore, based on these data, T_S_ should be used in conjunction with other well-described metrics of HS assessment (i.e., RR, core body temperature, feed intake, etc.) [2, 60].

**Fig. 2:**
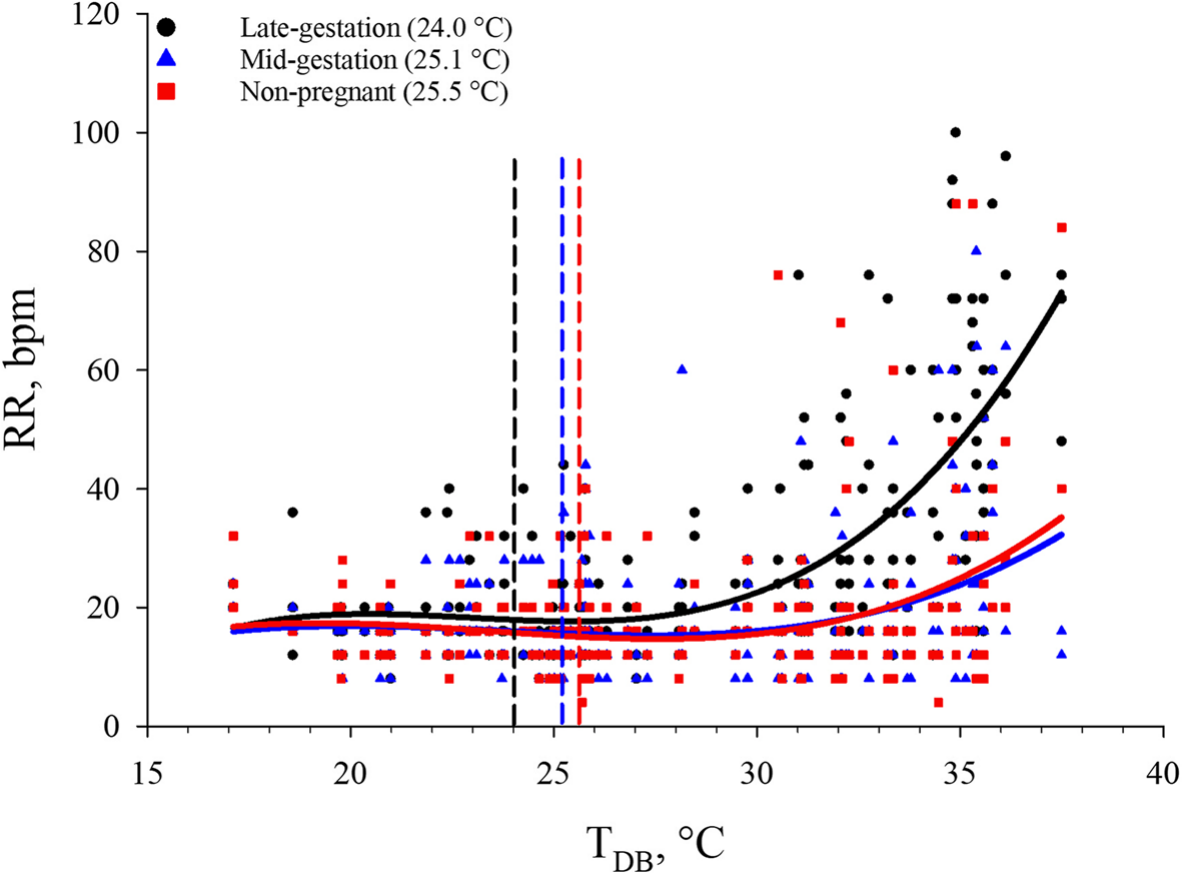
Effects of reproductive stage (late-gestation, mid-gestation, and non-pregnant) on respiration rate (RR) in multiparous sows (3.28 ± 0.81) exposed to incrementally increasing dry bulb temperature (T_DB_). Dashed lines indicate the inflection points and the T_DB_ associated with the inflection point is indicated in the legend. This T_DB_ was considered the point at which mild HS occurred

Evaporative heat loss via increased RR is an important method of thermoregulation for pigs as they do not possess functional sweat glands and must rely solely on heat loss through behavioral thermoregulation (i.e., wallowing, reduced feed intake, etc.) or via the skin and respiratory tract [2, 60, 72, 73]. Greater RR is often the first visual sign that pigs are suffering from HS and is considered an active form of heat loss by the sow and other species [13, 51, 52]. In the present study, the RR response to increasing T_DB_ at each reproductive stage was best described by cubic equations that differed by reproductive stage (*P* < 0.05; Table 1) and were used to calculate the RR inflection points (Fig. 2). The RR inflection point was considered the primary thermoregulatory indicator of mild HS as described in the mild HS equations (Table 4) and decision support tool (Fig. 5). The inflection points at which RR increased in response to increasing T_DB_ for non-pregnant, mid-gestation, and late-gestation sows were 25.5, 25.1, and 24.0 °C, respectively (Fig. 2). Although these data confirm previous reports that RR increases at lower T_DB_ in late-gestation versus mid-gestation and non-pregnant sows [31], they contradict previously calculated RR thresholds with increasing T_DB_. For example, when comparing RR to increasing T_DB_ in finisher pigs using a broken line assumption model, Huynh et al. [74] determined that the inflection point was 22 °C. It is important to note however that the finisher pigs in the aforementioned study [74] were fed ad libitum (as opposed to maintenance feeding common in non-pregnant and gestating sows) [43], likely increasing their metabolic heat production and resulting in greater thermal sensitivity to rising T_DB_. Furthermore, a recent review of 28 studies [75] reported an overall RR inflection point of 20 °C for pigs in response to increasing T_DB_. However, in this report [75], data were combined from prepubertal gilts, gestating sows, farrowing sows, lactating sows, and dry, non-pregnant sows to generate this value. Furthermore, no differentiation by production stage or physiological state (i.e., lactating sows are more HS sensitive than gestating and non-pregnant sows) was considered, which would likely result in an imprecise RR threshold estimation given the well-described thermoregulatory differences that exist by reproductive and production stage [29–31].

**Table 4:** Heat stress threshold equations to predict mild, moderate and severe heat stress in non-pregnant, mid-gestation, and late-gestation multiparous sows

Reproductive phase	Heat stress category	Equations
Non-pregnant	Mild	RR = 14.9527 + (–0.2009)(T_DB _– 25.5) + (0.07377)(T_DB_ – 25.5)^2^ + (0.005744)(T_DB_ – 25.5)^3^
	Moderate and severe	T_B_ = 38.1414 + (0.03256)(T_DB_ – 28.1) + (0.003542)(T_DB_ – 28.1)^2^ + (0.000264)(T_DB_ – 28.1)^3^
Mid-gestation	Mild	RR = 18.8330 + (0.1373)(T_DB_ – 25.1) + (–0.00238)(T_DB_ – 25.1)^2^ + (0.006759)(T_DB_ – 25.1)^3^ + (0.2227)(DP – 22.2) + (0.03973)(T_DB_ – 25.1)(DP – 22.2)
	Moderate and severe	T_B_ = 38.1325 + (0.05510)(T_DB_ – 27.8) + (0.001667)(T_DB_ – 27.8)^2^ + (0.001163)(DP – 18.0) + (0.002696)(T_DB_ – 27.8)(DP – 18.0)
Late-gestation	Mild	RR = 18.8849 + (0.02073)(T_DB_ – 24.0) + (0.02126)(T_DB_ – 24.0)^2^ + (0.02018)(T_DB_ – 24.0)^3^ + (0.1000)(DP – 19.1) + (0.02331)(T_DB_ – 24.0)(DP – 19.1)
	Moderate and severe	T_B_ = 38.1860 + (0.03922)(T_DB_ – 25.5) + (–0.00181)(T_DB_ – 25.5)^2^ + (0.000201)(T_DB_ – 25.5)^3^ + (0.01115)(DP – 17.8)

*RR*  Respiration rate, *T*_*DB*_ Dry bulb temperature, *T*_*B*_ Body temperature, *DP* Dew point

**Fig. 3:**
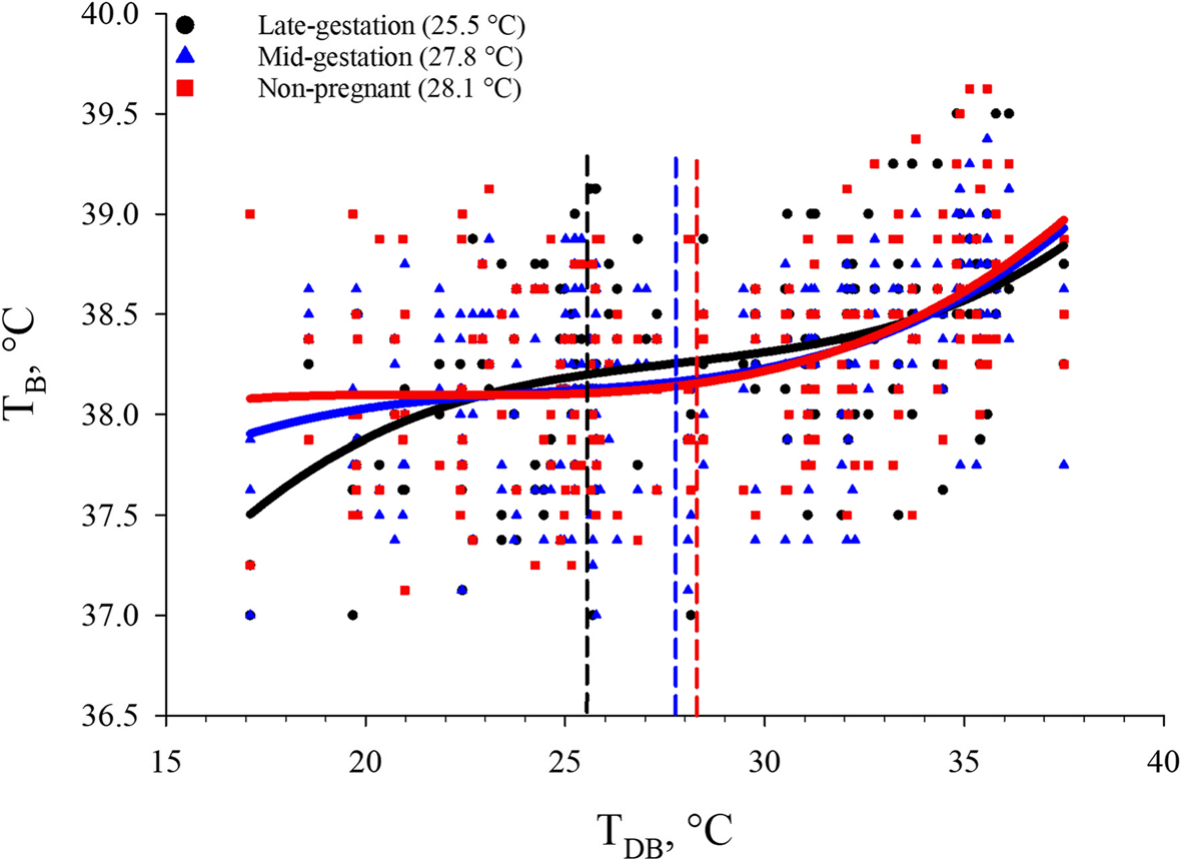
Effects of reproductive stage (late-gestation, mid-gestation, and non-pregnant) on body temperature (T_B_) in multiparous (3.28 ± 0.81) sows exposed to incrementally increasing dry bulb temperature (T_DB_) Dashed lines indicate the inflection points and the T_DB_ associated with the inflection point is indicated in the legend. This T_DB_ was considered the point at which moderate HS occurred

When heat gain from the environment overwhelms sensible and latent heat loss mechanisms, T_B_ will begin to rise above the euthermic T_B_ in response to increasing T_DB_ [13]. In the present study, it was determined that the T_B_ inflection point was best described by cubic equations that differed by reproductive stage (*P* < 0.05; Table 1). Body temperature inflection points for non-pregnant, mid-gestation, and late-gestation sows occurred at 0.10 °C above euthermic T_B_ and at a T_DB_ of 28.1, 27.8, and 25.5 °C, respectively (Fig. 3). The T_B_ inflection points for non-pregnant, mid-gestation, and late-gestation sows were used as the primary thermoregulatory indicator in the moderate HS equations (Table 4) and in the decision support tool (Fig. 5). This decrease in T_B_ inflection point with advancing reproductive stage was expected when considering the previously described increase in HS sensitivity as gestation advances [29–31, 34]. To our knowledge, this is the first study to describe the T_DB_ threshold at which heat loss mechanisms fail to allow sows at three reproductive stages with current genetics to maintain a euthermic T_B_. Although these T_DB_ inflection points cannot be considered the upper critical temperature (e.g., the point at which T_B_ begins to rise uncontrollably) [13], these data may provide a more precise T_DB_ threshold by which HS mitigation strategies should be employed in commercial swine facilities. Furthermore, it should be noted that the T_B_ inflection points at all reproductive stages were 3.9 to 6.5 °C less than what is currently described as the upper temperature extreme for sows > 100 kg (32 °C) [26].

**Fig. 4:**
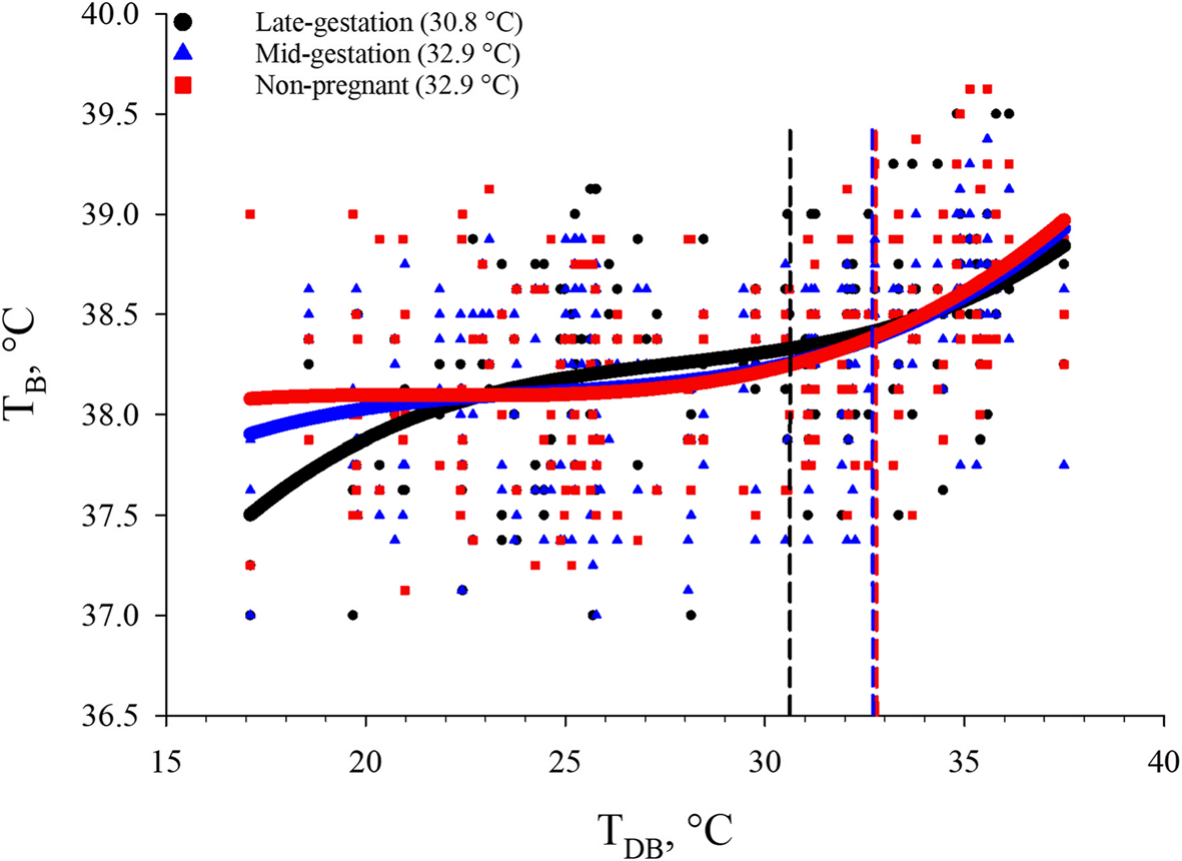
Effects of reproductive stage (late-gestation, mid-gestation, and non-pregnant) on body temperature (T_B_) in multiparous (3.28 ± 0.81) sows exposed to incrementally increasing dry bulb temperature (T_DB_). Dashed lines indicate the point at which T_B_ increased abruptly (+ 0.20 °C) above baseline T_B_ and the T_DB_ associated with this point is indicated in the legend. This T_DB_ was considered the point at which severe HS occurred

**Fig. 5:**
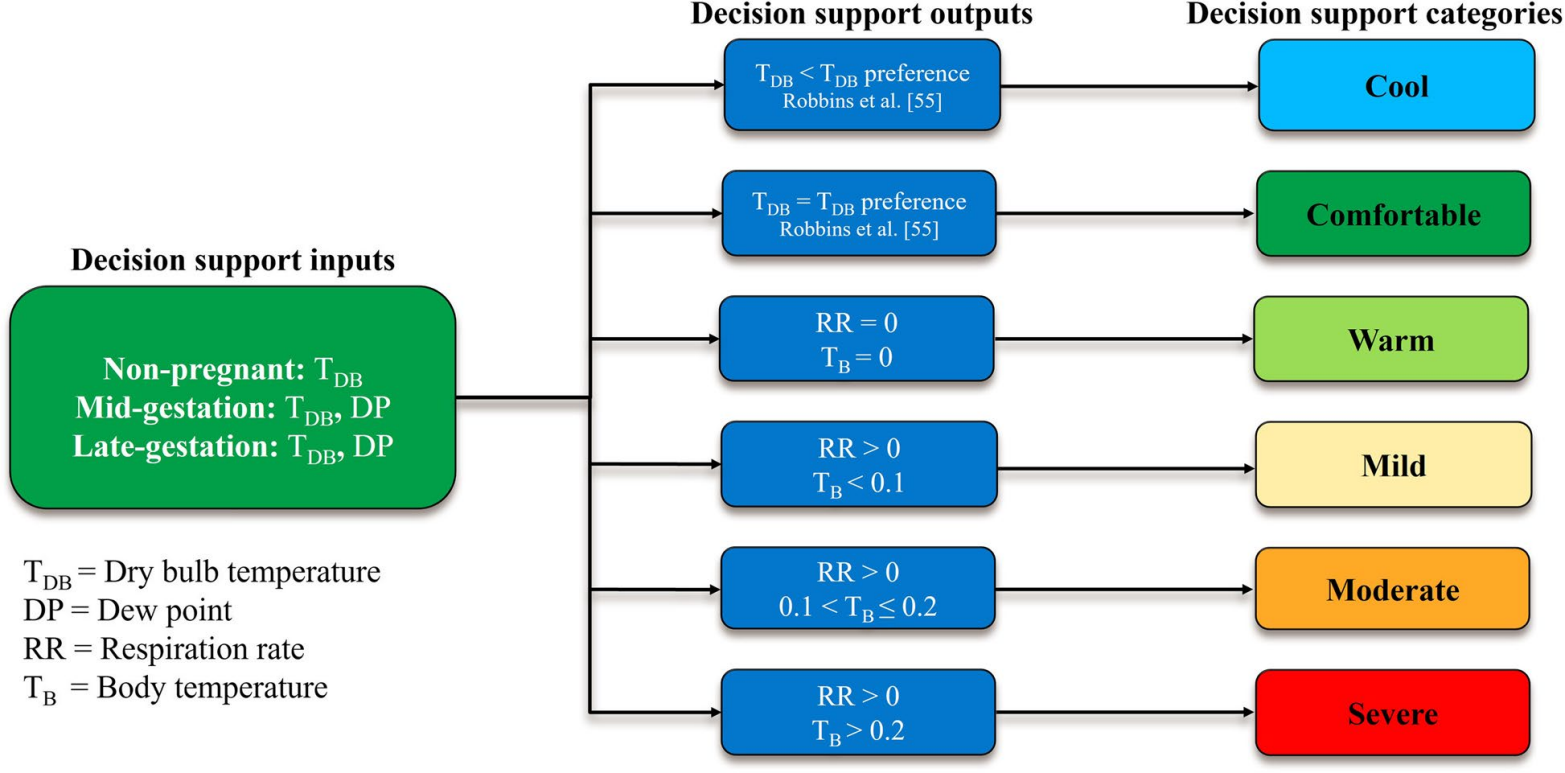
A behavior and physiology-based decision support tool designed to predict thermal comfort and stress in non-pregnant, mid-gestation, and late-gestation sows

An abrupt and uncontrolled T_B_ increase occurs when the upper critical temperature limit (UCT) has been reached [13]. In the present study, it was determined that the T_DB_ threshold at which the abrupt T_B_ increase occurred was at 0.20 °C above euthermic T_B_, and that it was similar (*P* > 0.05; 32.9 °C) for non-pregnant and mid-gestation sows, and lower (*P* < 0.05; 30.8 °C) for late-gestation versus non-pregnant and mid-gestation sows (Fig. 4). The T_B_ inflection points at which the abrupt T_B_ increase (0.20 °C) occurred for non-pregnant, mid-gestation, and late-gestation sows were used as the primary thermoregulatory indicator in the severe HS equations (Table 4) and in the decision support tool (Fig. 5). As expected, the severe HS threshold (e.g., the UCT) for late-gestation sows was deemed to be 1.2 °C lower than current guidelines for sows > 100 kg (32 °C) [26], and this response indicated that current UCT guidelines may not accurately reflect the severe HS threshold of late-gestation sows, which have greater heat gain and HS sensitivity when compared to non-pregnant and mid-gestation sows likely due to fetal growth [31]. However, the UCT for non-pregnant and mid-gestation sows in the present study was 0.90 °C greater than current > 100 kg sow guidelines (32 °C) [26]. While this response was unexpected given the aforementioned genetic advancements that have increased swine metabolic heat production [9, 76, 77] and likely sensitivity, this response may be explained by feed intake differences related to metabolic heat production and heat gain. In the present study and in commercial practice, gestating sows and non-pregnant sows in the breeding population are fed at maintenance to prevent excessive maternal weight gain [43]. This would likely lead to a decrease in heat production when compared to ad libitum fed populations as increased feed intake is associated with greater heat production due to the heat increment of feeding [13, 78]. Therefore, because current guidelines [26] do not differentiate by production stage, physiological state, or feeding level, it is likely that the UCT may be slightly greater for limit-fed sows > 100 kg based upon results from the present study (Fig. 4).

Relative humidity may play a role in the thermoregulatory response of pigs, particularly at greater T_DB_ [74]. For example, the efficacy of respiratory heat exchange with the environment is influenced by RH, and greater RH during HS causes increased RR in pigs [79]. In addition, increasing RH at greater T_DB_ leads to reductions in average daily body weight gain in grow-finish pigs [74]. As such, in addition to T_DB_, DP was incorporated in the development of the HS threshold equations (Table 4) for use in the decision support tool (Fig. 5). However, it was determined that the addition of DP was only significant (*P* < 0.05) for mid- and late-gestation sows and did not influence the RR or T_B_ response of non-pregnant sows (Table 1). This lack of a significant response to DP (*P* > 0.05) by non-pregnant sows may be due to their reduced HS sensitivity relative to mid- and late-gestation sows as we have previously reported [31]. Therefore, this may result in a decreased requirement for the activation of heat loss mechanisms and likely a reduced RR and T_B_ sensitivity to adverse climactic conditions in non-pregnant sows fed at maintenance.

Many thermal indices have used animal-based thermoregulatory responses to quantify HS intensity and often consider any T_DB_ below the HS threshold to be the T_DB_ range at which the species of interest is comfortable [80]. However, the absence of an active thermoregulatory (e.g., increased RR and T_S_) or T_B_ response is not necessarily an indicator of thermal comfort. This is because the transition from the thermal comfort zone to the warm zone is defined by limited thermoregulatory reactions defined by passive facilitation of heat loss that will intensify as T_DB_ increases [13]. As long as heat gain is balanced with heat loss, the animal is considered to be at thermoneutrality [81]; however, this T_DB_ range may not be reflective of an animal’s thermal comfort zone (e.g., the T_DB_ range in which an individual prefers to spend time in and feels relaxed) [80]. As such, the thermal preferences of non-pregnant, mid-gestation, and late-gestation sows were incorporated into the decision support tool (Fig. 5), and data were derived from a previous report by our group [55]. These data [55] indicate that non-pregnant and mid-gestation animals prefer a similar (*P* > 0.05) T_DB_ range (13.2 to 16.4 °C) while late-gestation sows prefer a slightly lower (*P* < 0.05) T_DB_ range (12.6 to 15.6 °C). Therefore, for utilization in the decision support tool (Fig. 5), the thermal comfort zone was defined as the T_DB_ range in which the sows prefer to spend most of their time. Additionally, based upon the thermal preference data, the cool zone was defined as any T_DB_ below the lower limit of the thermal comfort zone, and the warm zone was defined as the T_DB_ range in-between the upper limit of the thermal comfort zone and the start of mild HS (Fig. 2). Although maintaining facilities at sows’ thermal comfort zone may not be feasible during hotter times of the year or in regions with prolonged periods of HS, these guidelines may be useful during cooler times of the year when determining facility heating requirements.

## Conclusions

This study established HS thresholds and developed equations to predict mild, moderate, and severe HS in commercially relevant non-pregnant, mid-gestation, and late-gestation sows. These data were combined with thermal preference data previously reported by our group to develop a behavior and physiology-based decision support tool to predict thermal comfort and HS. Based on results from the present study, HS thresholds were influenced by reproductive stage and differed from previously established thresholds. In addition, the decision support tool developed through this research may be used to predict environmental conditions sows consider to be cool, comfortable, warm, mild HS, moderate HS, and severe HS. To our knowledge, this is the first thermal index developed specifically for gestating sows that incorporates both physiological and behavioral metrics of thermal preference and stress.

## Data Availability

The datasets used and/or analyzed during the current study are available from the corresponding author on reasonable request. All statistical codes used to analyze data are available as supplementary files.

## Abbreviations

AIC: Akaike's Information Criteria
B_0_: Intercept
B_1_: Linear regression coefficient
DP: Dew point
HS: Heat stress
IUHS: In utero heat stress
RH: Relative humidity
RR: Respiration rate
T_B_: Body temperature
T_DB_: Dry bulb temperature
THI: Thermal humidity index
TN: Thermoneutral
T_S_: Skin temperature
